# Whistling Lipoma: Bronchial Obstruction Caused by a Lipoma

**DOI:** 10.7759/cureus.7167

**Published:** 2020-03-03

**Authors:** Waqas Azhar, Fawwad Zaidi, Abdul Hannan

**Affiliations:** 1 Hospital Medicine, Springfield Clinic, Springfield, USA; 2 Internal Medicine, Memorial Medical Center, Springfield, USA; 3 Internal Medicine, Saint John Hospital, Springfield, USA; 4 Internal Medicine, Southern Illinois University School of Medicine, Springfield, USA; 5 Hematology/Oncology, Southern Illinois University School of Medicine, Springfield, USA

**Keywords:** lipoma, bronchial obstruction, bronchial neoplasm, airway obstruction, recurrent pneumonia

## Abstract

Lipomas are extremely common benign tumors that occur in a variety of locations. However, lipomas in the bronchus are exceptionally rare and account for a very small number of all bronchial tumors. Diagnosis of an endobronchial lipoma can be challenging at times, as they may present with overlapping symptoms of central airway obstruction, pneumonia, or dyspnea.

We present an 82-year-old male with a 40-pack-year history of smoking who presented with recurrent pneumonia and signs of airway obstruction. Imaging studies showed an obstructive lesion. The biopsy sample revealed adipose cells. Eventually, the lesion was successfully resected. The surgical specimen ruled out malignancy and confirmed the diagnosis of lipoma.

Timely identification and differentiating a malignant lesion from benign lesions, like lipoma, is crucial to management.

## Introduction

Tracheobronchial neoplasms are not very common. Malignant tumors account for most of the obstructive neoplasms in the tracheobronchial tree. Benign tumors arising from mesenchymal tissue are far less frequent [1-3]. Hamartomas and squamous cell papillomas are common compared to leiomyoma, lipoma, or mucous gland adenoma. These endobronchial tumors can cause symptoms of obstruction with recurring pneumonia or atelectasis, and hence, it is essential to identify and differentiate obstructive lesions of the airways in patients with recurrent pulmonary infections. Endobronchial lipomas are very rare and account for only 0.1% - 0.4% of all bronchial tumors. Lipomas grow slowly, symptoms are scarce if any, and usually, they remain undiagnosed for years until the onset of obstructive symptoms [1-5]. 

## Case presentation

**Figure 1:**
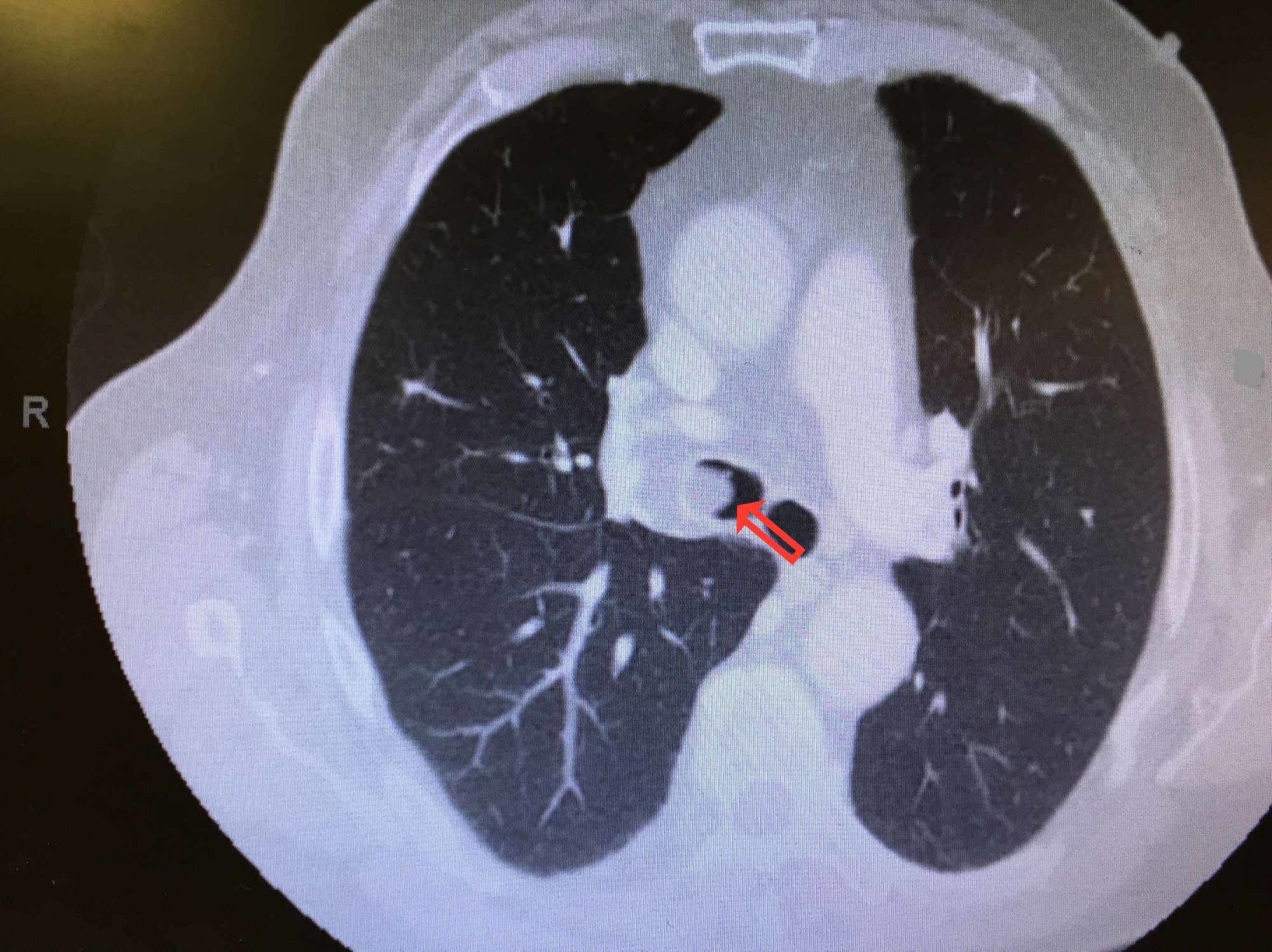
Computed tomography (CT) of the chest showing the obstructing lesion in the right mainstem bronchus

An 82-year-old Caucasian male with a 40-pack-year history of smoking, obesity, diabetes mellitus, hypertension, and three prior episodes of pneumonia within the last 12 months presented to the primary care physician with recurrence of his symptoms of dyspnea on exertion and wheezing. The patient was last treated for pneumonia four months ago. He denied fevers, orthopnea, paroxysmal nocturnal dyspnea, recent sick contact, or travel. He had a minimal non-productive cough. No weight loss was reported. The patient's vital signs, including resting oxygen saturation, were stable. Physical examination was remarkable for wheezing on the right side of his chest. He had no elevated jugular venous pulsation or lower extremity edema. No lymph nodes were palpable. Laboratory studies revealed normal blood counts, cardiac markers, and chemistry panel. Blood gases showed very mild compensated hypercapnia. A chest radiograph was done which revealed no new infiltrate but the resolution of the prior right-sided pneumonia. The patient was prescribed a short-acting bronchodilator, considering his long history of smoking. Upon follow-up evaluation, his symptoms did not improve and he had continued wheezing on the right side of the chest. A computed tomography (CT) scan of the chest was ordered, and the patient was referred to a pulmonologist. The CT scan showed a lesion in the right mainstem bronchus, predominantly fat density, most likely lipoma (Figure 1).

**Figure 2:**
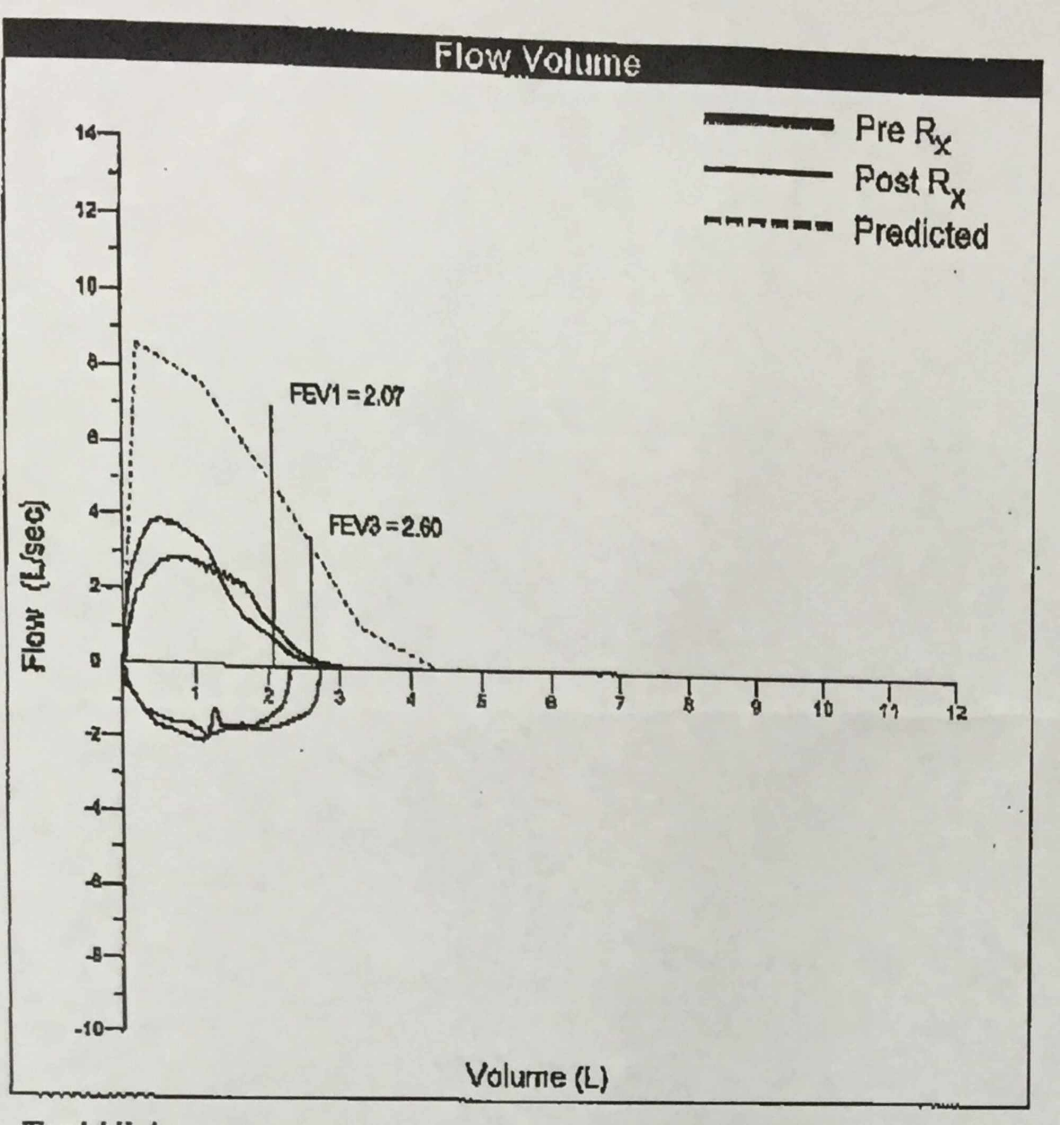
Flow volume loop showing fixed obstruction FEV1: forced expiratory volume in 1 second; FEV3: forced expiratory volume in 3 seconds; Rx: medical prescription

The pulmonologist performed a pulmonary function test, which revealed forced expiratory volume in 1 second (FEV1) of 67%, with mid-expiratory flow reduced to 42% of predicted and minimal, if any, reversibility with bronchodilators. Flow volume loops did show some signs of fixed obstruction (Figure 2).

**Figure 3:**
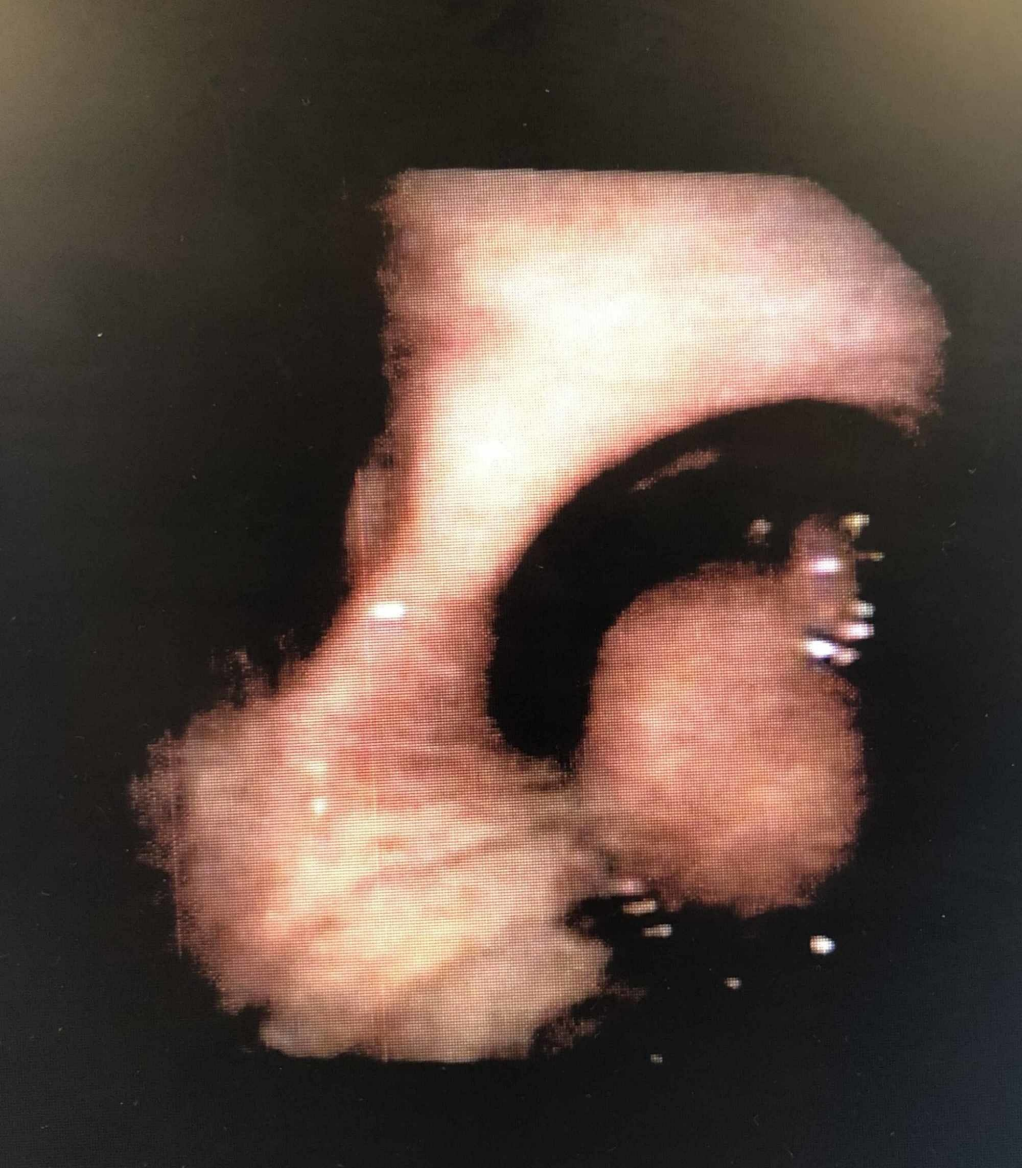
Bronchoscopy showing a lipoma in the right mainstem bronchus

He was scheduled for a bronchoscopy, which showed a nearly obstructed the right mainstem bronchus lesion (Figure 3).

**Figure 4:**
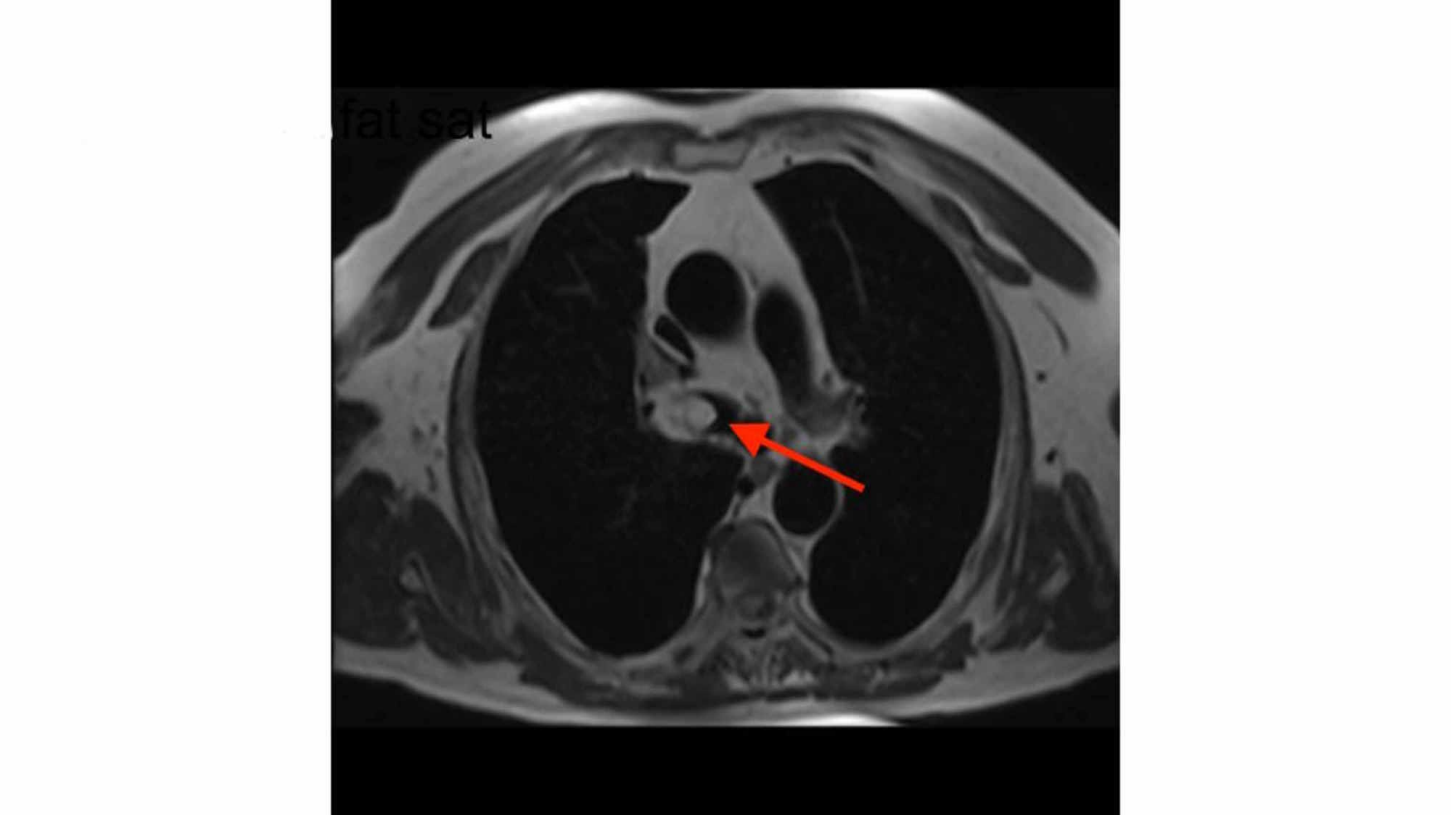
T2-weighted magnetic resonance imaging (MRI) shows fat density obstructive lesions in the right bronchus

The biopsy was difficult, but the sample revealed adipose cells. Magnetic resonance imaging (MRI) of the chest was performed which also confirmed fat density (Figure 4).

The scan also revealed a small defect within the lateral wall of the right mainstem bronchus with an extension of fat into the mediastinum, which limited the option of bronchoscopic resection. The patient underwent further mass resection, mediastinal dissection, and bronchoplasty. The surgical specimen confirmed lipoma with no evidence of malignancy. The patient did well postoperatively with a resolution of his wheezing. 

## Discussion

When a patient presents with recurrent pneumonia in a particular anatomical region, ruling out an anatomic abnormality or obstructive lesion is crucial. Obstruction or airway narrowing can be extrinsic from compression by neoplasms, lymphadenopathy (infective or neoplastic), and malformations involving big vessels or intrinsic caused by tracheomalacia, stenosis or strictures of the tracheobronchial tree, bronchial tumors, and occasionally foreign body impaction [6]. Malignant tracheobronchial tumors by far exceed benign tumors, which account for a tiny percentage [1-3]. An endobronchial lipoma is a very infrequently encountered benign neoplasm, usually appears in the fifth and sixth decade of life, and accounts for 0.1 - 0.4% of all bronchial tumors [4-5]. Lipomas are usually well-demarcated and soft. They mostly arise in the submucosal layer of the bronchial tree [4, 7]. Even though extensive studies are lacking, reported risk factors include a history of smoking and obesity [4-5].

Dyspnea, chest pain, cough, and post-obstructive pneumonia are the chief presenting signs and symptoms [8]. Lobar consolidation or atelectasis can be apparent on a chest radiograph, but bronchial neoplastic lesions are rarely detectable on plain chest radiographs [3, 9-10]. CT and MRI scans are pivotal in diagnosing the bronchial obstruction as they can precisely predict the fat density and differentiate fat tissue from other malignant or benign neoplastic lesions. The characteristic CT appearance of a lipoma is homogenous fat attenuation with approximately -100 Hounsfield units (HU) [9-10]. MRI further helps with accurately validating fatty tissue of the lesion [9].

Confirmation is achieved by biopsy or resection. Relieving obstruction and restoring the patency of the airway is the primary objective of therapy. Treatment modalities include endobronchial resection or surgical approaches. Less invasive, endobronchial techniques, like electrosurgical snaring and laser therapy, are the first choice for small lesions (< 4 cm) and proximal lesions with no evidence of extraluminal extension on imaging studies (CT and MRI) [4, 10-11]. Surgical methods are more appropriate when there is evidence of extraluminal spread, a high risk of malignancy, or if the lesions are distal and difficult to approach by bronchoscopy [10]. Patients with prior bronchial intervention and patients with high oxygen requirements should also be managed surgically [4].

## Conclusions

Endobronchial lipomas are rare tumors causing bronchial obstruction. Biopsy and resection are necessary in all cases, regardless of the symptoms, for timely identification and differentiation of malignant tumors from benign ones.
